# All-terrain vehicle exposure and the association of certified training on adolescent safety behaviors and crash experiences

**DOI:** 10.1186/s40621-022-00404-7

**Published:** 2022-12-21

**Authors:** Charles A. Jennissen, Katharine L. Champoux, Pamela J. Hoogerwerf, Kristel M. Wetjen, Lauren J. Mulford, Sienna E. Schaeffer, Uche E. Okoro, Gerene M. Denning

**Affiliations:** 1 Department of Emergency Medicine, Roy J. and Lucille A. Carver College of Medicine, University of Iowa, Iowa City, IA USA; 2 Stead Family Department of Pediatrics, Roy J. and Lucille A. Carver College of Medicine, University of Iowa, Iowa City, IA USA; 3 Roy J. and Lucille A. Carver College of Medicine, University of Iowa, Iowa City, IA USA; 4 Injury Prevention and Community Outreach, University of Iowa Stead Family Children’s Hospital, University of Iowa, Iowa City, IA USA; 5 Division of Pediatric Surgery, Roy J. and Lucille A. Carver College of Medicine, University of Iowa, Iowa City, IA USA

**Keywords:** All-terrain vehicles, Adolescents, Certification course, Crash, Helmets, Injury, Passengers, Pediatrics, Public roads, Training

## Abstract

**Background:**

Certified training courses in all-terrain vehicle (ATV) operation are recommended, but little has been published regarding how they affect riding behaviors. Our objectives were to determine adolescents’ ATV riding exposures, crashes and injuries, and the association of completing certified ATV training on riding behaviors.

**Methods:**

Respondents completed an anonymous paper survey at 30 school districts approximately one year after participating in the Safety Tips for ATV Riders (STARs) program. Survey data were compiled using Qualtrics™. Frequency, chi-square, Fisher’s exact test, and logistic regression analyses were performed using SAS, V.9.4.

**Results:**

4,891 students completed the survey from Fall 2012–Fall 2019. Respondents were 10–18 years old, and similar numbers of participants were male and female. Fifty-nine percent lived in town, 18% on a farm, and 22% in the country but not on farm. Forty-two percent reported their families owned an ATV. Seventy-seven percent had ridden an ATV, 40% of whom reported riding at least weekly. The vast majority (94%) used ATVs for recreation and 49% used them for work purposes. In the previous year, 22% of riders reported having been in a crash, with 7% of crash victims requiring medical attention for injuries. Greater crash likelihood was seen in males, recreational riders, more frequent riders, competitive racers and those who had ridden with passengers or on roads. Only 8% of riders had completed certified ATV safety training (note, STARs is not a certification program). Those whose families owned ATVs, more frequent riders, and public park users had greater likelihoods of course completion. Relative to their peers, respondents who completed a certification course had higher proportions that always/almost always wore helmets (39% vs. 20%, *p* < 0.0001) and lower proportions that never/almost never wore helmets (29% vs 58%, *p* < 0.0001), had ridden with passengers (63% vs. 96%, *p* < 0.0001), and had driven on public roads (41% vs 50%, *p* = 0.0065).

**Conclusions:**

ATV safety training certification among Iowa adolescents in the study was infrequent but those that received training reported higher helmet use, less riding with passengers, and less driving on public roads. These data suggest completing safety training certification may promote safer riding behaviors among youth.

## Background

All-terrain vehicles (ATVs) continue to be a significant threat for injuries and deaths in children and adolescents (Denning and Jennissen 2018). Of the approximately 100,000 annual ED visits for ATV-related injuries in the United States, over a quarter of patients are younger than 16 years old, and this age group has accounted for more than one-fifth of all deaths due to ATVs from 1982 to 2018 (3,353 of 15,744) (Topping 2018). In fact, more children less than 16 years of age in the United States die from ATVs each year than from bicycle crashes (Helmkamp et al. 2009). Moreover, a national study of all pediatric ATV-related deaths found 16- to 17-year-olds had 27% more fatalities than youth < 16 years of age (Denning et al. 2014).

The American Academy of Pediatrics recommends that no child less than 16 years of age should ride an ATV (American Academy of Pediatrics Committee on Injury and Poison Prevention 2000). However, many parents are unaware of or ignore this guidance. One statewide study of young adolescents found that more than three-quarters had ridden an ATV (Jennissen et al. 2014), and more than 95% have reported doing so in other studies surveying primarily rural youth (Burgus et al. 2009; Campbell et al. 2010; Hafner et al. 2010). Although rural populations have a greater likelihood and higher frequency of riding ATVs (Jennissen et al. 2014), children and teenagers in urban areas are not immune to ATV exposure and crashes (Jennissen et al. 2014; Qin et al. 2017). More than 1 in 5 urban youth stated they had ridden at ATV in a US YouthStyles survey (Shults and West 2015).

Training and education are mainstays of public health disease and injury prevention and are considered essential strategies in decreasing ATV-related injuries and deaths (Jennissen et al. 2015). However, survey studies have found that only 15–26% of youth ATV riders have received any safety education or training (Burgus et al. 2009; Campbell et al. 2010; Jones and Bleeker 2005). Less than half of Illinois 4-Hers recalled hearing any information regarding ATV safety, and most who did had received it from television or newspapers (Hafner et al. 2010). Even fewer adolescents, as low as 1–5%, have completed an ATV certification course with hands-on training (Campbell et al. 2010; Tormoehlen and Sheldon 1996). Most riders report being largely self-taught or having minimal instruction from a relative or peer (Rodgers 1999; Aitken et al. 2004).

Children and teenagers frequently fail to follow safety rules while riding on ATVs (Jennissen et al. 2014). These rules include no passengers (almost all vehicles are designed for an operator only), no riding on public roads, and always wearing a helmet (Jennissen et al. 2014). Proper safety behaviors are taught to students in certified ATV training, but there is little published data regarding how training affects subsequent riding behaviors. Our study objectives were to determine adolescent’s exposure to ATVs, riding behaviors, and crash and injury experiences. In particular, we wanted to determine factors related to having completed an ATV certification training course and its association with subsequent riding behaviors.

## Methods

### Study population

**Fig. 1:**
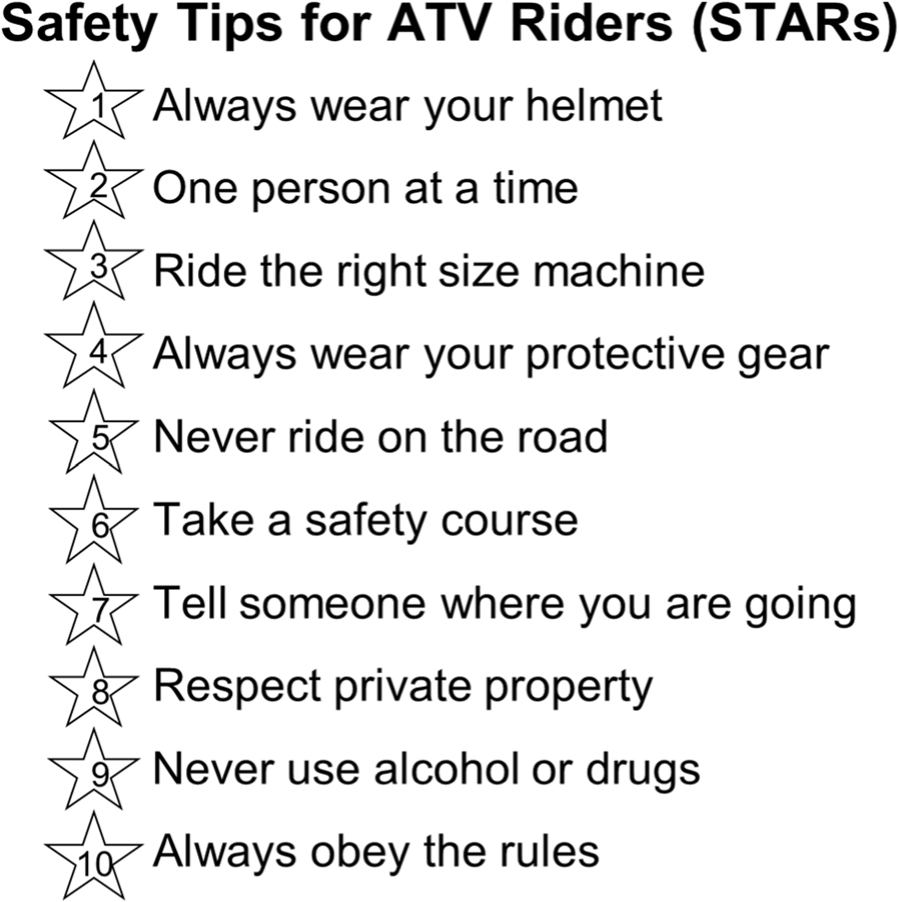
The ten safety tips presented in the Safety Tips for ATV Riders (STARS) program

A written survey was administered to Iowa children and adolescents in their classrooms about one year after they had participated in the Safety Tips for ATV Riders (STARs) program at their school. All participants were unique and completed the STARs program and the follow-up survey only once. The STARs program is an interactive educational intervention that increases students’ awareness of the key principles of safe ATV operation which are highlighted by the program’s ten STARs (Jennissen et al. 2015). See Fig. 1. However, the program is not an ATV safety training certification course. Participating schools were recruited through communication with school nurses and administrators. In many cases, multiple grades participated in the STARs program when it was given at a school. One year after the STARs program, paper surveys were mailed to the schools and distributed in classrooms with students that had participated in the program. These were then mailed back in self-addressed, postage-paid envelopes by the schools. Surveys were completed from Fall 2012–Fall 2019 by students 10–18 years of age (grades 5–12) at 30 Iowa school districts. There were no changes in Iowa law regarding ATV use by children and adolescents during the study period.

### Survey

The survey was developed through a collaborative and iterative process by members of the Iowa Off-Road Vehicle Task Force which is led by the University of Iowa Stead Family Children’s Hospital’s Injury Prevention Program. The Task Force is composed of a multidisciplinary group of dedicated individuals, community organizations, and various stakeholders concerned about pediatric ATV injuries and deaths in the state of Iowa.

The survey was administered to ten adolescents with ties to the Task Force members. Participants were asked to explain and clarify their responses if a question was not easily understood. The youth’s written and verbal responses were compared for consistency. These results were then utilized to inform the final survey design.

The survey was first distributed in Fall 2012 to schools that had participated in the STARs program in Fall 2011. Demographic variables included in the survey were sex, age, and residence type (e.g., on a farm). Other questions included whether they had ever: ridden an ATV, participated in organized ATV racing, ridden in an off-highway vehicle (OHV) park, and/or completed an ATV training certification course. Students were also asked if their family owned an ATV and how they personally used ATVs (for work, for recreation, or both). In 2014, the survey was revised to include questions regarding their frequency of ATV use, whether they had experienced an ATV crash or required medical attention for an ATV crash-related injury, and their riding behaviors (helmet use frequency, riding with passengers, and riding on public roads) in the previous 12 months.

### Data analysis

The STARs program’s completed surveys were provided to the primary investigator of this study for analysis. The authors’ Institutional Review Board deemed the study exempt as the research analysis was done on an existing dataset that had been collected anonymously with no personal identifiers. Responses of the paper surveys were entered into Qualtrics™, a web-based survey tool. Data were then exported as an Excel spreadsheet and imported into the statistical software suite, SAS (previously “Statistical Analysis System”), version 9.4 (SAS Institute, Cary, NC). Descriptive (frequencies), bivariate (chi-square, Fisher’s exact test), and multivariable logistic regression analyses were performed. Multiple regression was done controlling for all covariates and results reported as adjusted odds ratios. Particular attention was performed analyzing factors regarding having completed an ATV training certification course and the association of having completed a course on riding behaviors. All *p* values were two-tailed, and a value < 0.05 was considered statistically significant. Missing data were not included in analyses.

## Results

### Demographics and ATV riding characteristics

A total of 4,891 Iowa students completed the survey with 2,706 of these completing the revised version that included questions on ATV riding frequency, ATV crash and crash-related injury, and riding behaviors in the previous 12 months. The population was equal by sex with three-quarters (76%) being 12–14 years old. See Table 1. Nearly three-fifths lived in a town (59%), and one-fifth lived on a farm (18%) or resided in the country but not on a farm (22%). Over two-fifths (42%) of the student’s families owned an ATV, and over three-quarters had ridden on an ATV (77%). Only 8% of riders had completed an ATV training certification course (the STARs program is not a certification course).

**Table 1:** Overall demographics and variable frequencies of school-aged children that completed questions regarding all-terrain vehicles (ATVs) on the Safety Tips for ATV Riders (STARs) one-year follow-up survey

	Total (*N* = 4,891)
Variable	*n* (Col%)^a^
Sex (*n* = 4849)	
Male	2460 (51)
Female	2389 (49)
Age (*n* = 4783)	
< 11 yrs	88 (2)
11 yrs	374 (8)
12 yrs	973 (20)
13 yrs	1463 (31)
14 yrs	1176 (25)
15 yrs	562 (12)
≥ 16 yrs	147 (3)
Where they live (*n* = 4865)	
On a farm	898 (18)
In country/not farm	1087 (22)
In town	2880 (59)
Family owns an ATV (*n* = 4861)	
Yes	2041 (42)
No	2820 (58)
Ridden an ATV ever (*n* = 4891)	
Yes	3766 (77)
No	1125 (23)
ATV training certification course completed (*n* = 3747) ^b^	
Yes	303 (8)
No	3444 (92)
Competitive ATV racing (*n* = 3721) ^b^	
Yes	317 (9)
No	3404 (91)
Ridden in a public OHV park (*n* = 3729) ^b^	
Yes	548 (15)
No	3181 (85)
Frequency of ATV use past 12 months (*n* = 2704)^c^	
Almost daily	458 (17)
About once a week	624 (23)
About once a month	598 (22)
Only a few times	1024 (38)
Had an ATV crash in past 12 months (*n* = 2706)^c^	
Yes	590 (22)
No	2116 (78)
Required medical attention for an ATV crash injury in past 12 months (*n* = 590)^d^	
Yes	42 (7)
No	548 (93)

*ATV* all-terrain vehicle; *Col%* column percent; *MV* motor vehicle; *OHV* off-highway vehicle

^a^Column total may not equal group *N* due to missing data

^b^Of those that had ever been on an ATV

^c^Of those who had been on an ATV in past 12 months

^d^Of those who had been in an ATV crash

Nine percent of students reported that they had participated in organized competitive ATV racing and 15% had ridden in a public off-highway vehicle (OHV) park. Of those that completed the revised version of the survey and reported riding an ATV in the past 12 months, two-fifths reported riding an ATV at least weekly. Over one-fifth (22%) of ATV riders reported they had been in an ATV crash in the past year. A crash was defined as hitting something, being ejected from the vehicle, or rolling over. Of those in a crash, 7% required medical attention for an ATV-related injury.

### Occupational versus recreational use

About half (51%) of riders used ATVs for recreation only, while 43% reported riding for both work and recreation, and 6% stated they rode ATVs for work purposes only. See Table 2. Males, students that lived on farms, and those whose families owned ATVs all had higher proportions that used ATVs at least sometimes for work purposes on cross-tab analyses (all *p* < 0.0001). Logistic regression analysis showed that males were nearly twice as likely as females to use ATVs for work-related purposes. Similarly, those living on farms were over four times more likely than students living in town, and students whose families owned ATVs were over 3.5 times more likely than those who did not to report using ATVs for work.

**Table 2:** Cross-tab and logistic regression analyses of how ATVs are used by demographics for those school-aged children that had been on an ATV in the past 12 months and completed the Safety Tips for ATV Riders (STARs) program’s one-year follow-up survey

Variable	ATV use				Logistic regression analysis	
	Work only (*N* = 221, 6%)	Both (*N* = 1586, 43%)	Recreation only (*N* = 1905, 51%)		For work only/both vs. recreation only (*N* = 3520)	
	*n* (Row%)^a^	*n* (Row%)^a^	*n* (Row%)^a^	P value	aOR^b^	95% CI
Sex (*n* = 3674)						
Male	126 (7)	940 (49)	852 (44)	< 0.0001	1.91	1.64–2.22
Female	93 (5)	638 (36)	1025 (58)		1.00 (ref)	
Age (*n* = 3623)						
≤ 11 yrs	24 (7)	140 (42)	168 (51)	0.12	1.00 (ref)	
12–13 yrs	113 (6)	734 (40)	970 (53)		0.71	0.53–0.96
14–15 yrs	75 (6)	617 (46)	664 (49)		0.87	0.65–1.17
≥ 16 yrs	5 (4)	49 (42)	64 (54)		0.72	0.44–1.16
Where they live (*n* = 3682)						
On a farm	83 (10)	563 (69)	165 (20)	< 0.0001	4.29	3.46–5.33
In country/not farm	36 (4)	384 (43)	472 (53)		1.11	0.92–1.32
In town	100 (5)	626 (32)	1253 (63)		1.00 (ref)	
Family owns an ATV (*n* = 3687)						
Yes	123 (5)	1172 (59)	677 (34)	< 0.0001	3.72	3.17–4.35
No	97 (6)	398 (23)	1220 (71)		1.00 (ref)	

*aOR* adjusted odds ratio; *ATV* all-terrain vehicle; *CI* confidence interval; *Row%* row percent, *yrs* years

^a^Column total may not equal group *N* due to missing data

^b^Controlling for all other variables in the table

### Certified ATV training course completion

As previously noted, of those that had been on an ATV, only 8% had taken an ATV training certification course. On cross-tab analysis, a number of groups had significantly higher proportions that had taken a course. See Table 3. These groups included males, those whose family owned an ATV, more frequent riders (at least weekly), those who performed work with ATVs, students that reported having ridden in a public OHV park, and organized competitive racers (*p* = 0.012 for males vs females, all other comparisons *p* < 0.001). Logistic regression analysis showed those living on farms were 0.6 times less likely to have taken a certification course than their peers. In addition, students whose families owned an ATV were over 1.5 times more likely than non-owners, frequent riders (at least weekly) were 1.5 times more likely than less frequent riders, and public OHV park users were about twice as likely as non-users to have completed a course.

**Table 3:** Cross-tab and logistic regression analysis of having ever completed a certified ATV training course by demographics and other variables for those school-aged children that had been on an ATV in the past 12 months and completed the Safety Tips for ATV Riders (STARs) program one-year follow-up survey

Variable	Completed training course			Logistic regression	
	Yes (*N* = 303)	No (*N* = 3444)		Analysis (*N* = 2573)	
	*n* (Row%)^a^	*n* (Row%)^a^	P Value	aOR^b^	95% CI
Sex (*n* = 3709)					
Male	178 (9)	1751 (91)	0.012	1.14	0.86–1.50
Female	124 (7)	1656 (93)		1.00 (ref)	
Age (*n* = 3654)					
≤ 11 yrs	34 (10)	302 (90)	0.055^c^	1.00 (ref)	
12–13 yrs	159 (9)	1667 (91)		1.00	0.60–1.68
14–15 yrs	101 (7)	1272 (93)		0.76	0.44–1.30
≥ 16 yrs	4 (3)	115 (97)		0.40	0.13–1.24
Where They Live (*n* = 3717)					
On a farm	69 (8)	746 (92)	0.77	0.61	0.42–0.88
In country/not farm	68 (8)	835 (92)		0.82	0.58–1.15
In town	158 (8)	1841 (92)		1.00 (ref)	
Family owns an ATV (*n* = 3722)					
Yes	212 (11)	1773 (89)	< 0.0001	1.55	1.08–2.23
No	87 (5)	1650 (95)		1.00 (ref)	
How they used ATVs (*n* = 3696)					
Work only	23 (10)	197 (90)	< 0.0001	1.00 (ref)	
Both work and recreation	162 (10)	1418 (90)		0.61	0.36–1.03
Recreation only	118 (6)	1778 (94)		0.61	0.5–1.06
Frequency of ATV use in past 12 months (*n* = 2693)					
At least weekly	149 (14)	929 (86)	< 0.0001	1.53	1.11–2.11
Monthly or less	112 (7)	1503 (93)		1.00 (ref)	
Organized competitive racing participation (*n* = 3701)					
Yes	59 (19)	257 (81)	< 0.0001	1.41	0.95–2.10
No	240 (7)	3145 (93)		1.00 (ref)	
Ridden in a public OHV park (*n* = 3704)					
Yes	92 (17)	453 (83)	< 0.0001	1.99	1.44–2.75
No	211 (7)	2955 (93)		1.00 (ref)	

*aOR* adjusted odds ratio; *ATV* all-terrain vehicle; *CI* confidence interval; *OHV* off-highway vehicle; *Row%* row percent, *yrs* years

^a^Column total may not equal group *N* due to missing data

^b^Controlling for all other variables in the table

^c^Fisher’s Exact Test used as values in the analysis < 5

### Certified ATV training course completion and riding behaviors

Students who had completed a certified ATV training course reported more safe riding behaviors than those that had not completed training. See Table 4. Trained riders had higher proportions that always or almost always wore their helmets (39% vs. 20%, *p* < 0.0001) and lower proportions that never or almost never wore a helmet (29% vs. 58%, *p* < 0.001). Certified riders also had lower percentages reporting riding as or with passengers (63% vs. 96%, *p* < 0.0001) and of riding on public roads (41% vs. 50%, *p* = 0.0065).

**Table 4:** Cross-tab analysis of having ever completed a certified ATV training course by safety behaviors in the past 12 months for those school-aged children that had been on an ATV in the past 12 months and completed the revised Safety Tips for ATV Riders (STARs) program one-year follow-up survey

Variable	Completed training course		
	Yes (*N* = 303, 8%)	No (*N* = 3444, 92%)	
	*n* (Col%)^a^	*n* (Col%)^a^	P Value
Helmet use frequency (*n* = 2693)			
Always or almost always	103 (39)	497 (20)	< 0.0001
More than half the time	39 (15)	231 (10)	
Less than half the time	45 (17)	296 (12)	
Never or almost never	76 (29)	1406 (58)	
Ridden with passengers (*n* = 2686)			
Yes	164 (63)	1910 (96)	< 0.0001
No	97 (37)	515 (4)	
Ridden on public roads (*n* = 2684)			
Yes	108 (41)	1208 (50)	0.0065
No	155 (59)	1213 (50)	

*ATV* all-terrain vehicle; *Col%* column percent

^a^Column total may not equal group *N* due to missing data

### ATV crashes in the past year

Overall, over one-fifth of riders had been in at least one ATV crash in the past 12 months. See Table 5. On cross-tab analysis, youth with higher proportions having had a crash included males, those living on farms, students whose families owned an ATV, more frequent ATV users, those who used ATVs for recreation, students who reported having participated in organized ATV racing, and those reporting having ridden with passengers and riding on public roads (all *p* = 0.0017 or less). Logistic regression analysis showed males and those using ATVs for recreation were both about twice as likely to have been in a crash as compared to females and those who used them for work only, respectively. As compared to those who rode ATVs several times a year or less, more frequent riders were incrementally more likely to be in a crash with almost daily riders being over 4 times more likely to have been in a crash in the past year. Students reporting that they had participated in organized ATV racing were more than three times as likely as non-racers to report having been in at least one ATV crash. Those reporting unsafe riding behaviors such as riding with passengers and riding on public roads were both over 1.5 times more likely to have been in a crash than riders who avoided these unsafe practices. Although riders who had completed an ATV training certificate course had higher proportions who had been in a crash by cross-tab analysis, there was no significant difference found after performing logistic regression analysis and controlling for other variables.

**Table 5:** Cross-tab and logistic regression analyses of having had an ATV crash in the past 12 months by demographics and other variables for those school-aged children that had been on an ATV in the past 12 months and completed the Safety Tips for ATV Riders (STARs) program one-year follow-up survey

Variable	ATV Crash in Past 12 Months			Logistic regression analysis	
	Yes (*N* = 590)	No (*N* = 2706)		(*N* = 2498)	
	*n* (Row%)^a^	*n* (Row%)^a^	P Value	aOR^b^	95% CI
Sex (*n* = 2680)					
Male	400 (28)	1038 (72)	< 0.0001	1.81	01.44–2.25
Female	184 (15)	1058 (85)		1.00 (ref)	
Age (*n* = 2648)					
≤ 11 yrs	53 (22)	192 (78)	0.75	1.00 (ref)	
12–13 yrs	306 (23)	1050 (77)		0.89	0.61–1.30
14–15 yrs	198 (21)	761 (79)		0.70	0.48–1.04
≥ 16 yrs	19 (22)	69 (78)		0.72	0.37–1.41
Where they live (*n* = 2683)					
On a farm	183 (26)	519 (74)	0.0017	0.92	0.69–1.22
In country/not farm	152 (22)	536 (78)		1.01	0.77–1.32
In town	248 (19)	1045 (81)		1.00 (ref)	
Family Owns an ATV (*n* = 2688)					
Yes	446 (26)	1270 (74)	< 0.0001	0.98	0.74–1.31
No	140 (14)	832 (86)		1.00 (ref)	
How they used ATVs (*n* = 2691)					
Work only	16 (7)	214 (93)	< 0.0001	1.00 (ref)	
Recreation only	214 (19)	933 (81)		2.45	1.36–4.41
Both work and recreation	357 (26)	1024 (74)		1.89	1.06–3.35
Frequency of ATV use in past 12 months (*n* = 2688)					
Almost daily	205 (45)	250 (55)	< 0.0001	4.34	3.02–6.23
About once a week	174 (28)	447 (72)		2.57	1.85–3.58
About once a month	104 (18)	489 (82)		1.52	1.09–2.11
Several times a year or less	103 (10)	916 (90)		1.00 (ref)	
Organized competitive racing participation (*n* = 2692)					
Yes	150 (55)	123 (45)	< 0.0001	3.28	2.44–4.40
No	437 (18)	1982 (82)		1.00 (ref)	
Ridden with Passengers (*n* = 2686)					
Yes	511 (25)	1563 (75)	< 0.0001	1.84	1.36–2.48
No	77 (13)	535 (87)		1.00 (ref)	
Ridden on public roads (*n* = 2683)					
Yes	402 (31)	914 (69)	< 0.0001	1.74	1.37–2.20
No	183 (13)	1184 (87)		1.00 (ref)	
ATV certification training course completed (*n* = 2695)					
Yes	81 (31)	181 (69)	0.0002	1.38	0.99–1.93
No	506 (21)	1927 (79)		1.00 (ref)	

*aOR* adjusted odds ratio; *ATV* all-terrain vehicle; *CI* confidence interval; *Row%* row percent; *yrs* years

^a^Column total may not equal group *N* due to missing data

^b^Controlling for all other variables in the table

## Discussion

Three-quarters of Iowa adolescents in our study reported having ridden on an ATV. Of these, 40% reported riding at least weekly and 62% at least monthly. Although most youth used ATVs for recreation (94%), nearly half of study participants also used them for work-related purposes. Crashes were common with more than one-fifth of adolescents having had at least one crash in the previous 12 months and 7% of these individuals requiring medical attention for an injury. Formal ATV training through a certification course was rare, but we found that those that had received this type of training reported safer riding behaviors.

In the past, dealers often offered free ATV safety training when selling a vehicle, but rider participation in such programs was generally low (i.e., no greater than 11%) (Rodgers 1999). A study of ATV owning households found that nearly a third of those who recalled being offered dealer training did not accept the offer because they “already knew how to ride” (Rodgers 1999). Similarly, among FFA (formerly Future Farmers of America) members at a national convention who indicated they were not interested in training, about a quarter of the teenagers “felt” they did not need training and an additional 16% reported they were already safe drivers (Burgus et al. 2009).

Consistent with this lack of interest in training, we found that only 8% of ATV riders in our study reported having completed a certified ATV training course. It is best for courses to include hands-on experiences like those conducted by ATV Safety Institute instructors (ATV Safety Institute 2021). This training allows the opportunity for students to practice riding skills in a controlled and supervised environment. However, many ATV safety certification courses are now through online education only, such as that offered at ATVcourse.com by a number of state departmental organizations (Fresh Air Educators Inc. 2021). The Iowa Department of Natural Resources is one of the eleven state organizations that has an approved course at ATVcourse.com. The study survey did not ask students whether the certification course they had completed included hands-on training or not. Other studies that have specifically asked about completion of an ATV certification course with hands-on training have found even lower percentages than we did (Campbell et al. 2010; Tormoehlen and Sheldon 1996).

Despite a general disinterest in training, a survey study of youth found that most knew that safe use of an ATV should include taking a hands-on riding course (Williams et al. 2011). Moreover, a focus group study of teenagers found that they acknowledged a responsibility to be educated on ATV safety and overall would accept training requirements (Aitken et al. 2004). However, few states have ATV training certificate or licensing requirements (Specialty Vehicle Institute of America 2021). Many of those do only mandate such education if the adolescent is riding on public not private lands which significantly limits their effectiveness (Denning et al. 2013).

The 10-Year Consent Decree between ATV manufacturers and the US Consumer Product Safety Commission had provisions related to education and training that were shown to increase safer riding behaviors and decrease crash risk (Rodgers 1993). Likewise, studies of youth have found that those who had received ATV safety training had lower percentages reporting a crash than those with no training (Hafner et al. 2010) and had safer riding behaviors such as increased helmet use (Burgus et al. 2009).

In our study, we wanted to see if there were any differences between adolescents that had completed an ATV training certification course and those that did not. We found significant differences in three major riding behaviors that, if not followed, increase the risk for ATV-related crash and/or injury. Youth that had completed an ATV training certification course had higher proportions that always/almost always wore a helmet and lower proportions that never/almost never wore a helmet, rode with or as a passenger, or traveled on public roads. Although students that had completed training certification had higher percentages that had been in an ATV crash, these differences were not significant when other variables were taken into account.

## Limitations

Our study is limited in that it was performed in a single Midwest state with a primarily White, non-Hispanic population, and the subjects were a convenience sample of students from schools participating in the STARs program rather than a randomized sample. Therefore, our results may not be generalizable to other areas of the country or even the entire state. As discussed earlier, we did not question subjects as to whether the certification course they had completed included hands-on training and personal interaction with an instructor or was entirely online. Like other survey studies, our data may be subject to bias because of inaccurate recall or social desirability bias. The latter should have been decreased by the collection of data anonymously.

## Conclusions

Iowa adolescents in the study had high exposures to ATVs and crashes were common. The vast majority reported using ATVs for recreation, but almost half of the subjects also used them for work. ATV training certification course completion by youth riders was rare. However, those that received training did report safer riding behaviors including greater helmet use, less riding with passengers, and less driving on roads as compared to untrained riders. For youth who ride ATVs despite AAP recommendations against it, multi-targeted approaches including education/training and enforced safety regulations are needed to increase safe riding behaviors and decrease ATV-related deaths and injuries. In addition, all youth should complete formal safety training, preferably with a hands-on component, before operating an ATV.

## Data Availability

Data and materials are available to other parties for research purposes after a data sharing agreement plan is agreed to and signed. Those interested should contact the corresponding author.

## Abbreviations

ATV: All-terrain vehicle
e.g.: Exempli gratia (for example)
OHV: Off-highway vehicle
i.e.: Id est (that is)
SAS: Previously stood for statistical analysis system
STARs: Safety Tips for ATV Riders
TM: Trademark
US: United States
